# Free tissue transfer for trophic ulcer complicating leprosy

**DOI:** 10.4103/0970-0358.53022

**Published:** 2009

**Authors:** Yogesh C. Bhatt, Nikhil S. Panse, Kinnari A. Vyas, Gunjan A. Patel

**Affiliations:** Department of Plastic Surgery, SSG Hospital and Medical College, Baroda, India

**Keywords:** Free flap, Post Hansen's trophic ulcer, Nonsensate foot

## Abstract

Plantar ulceration is the commonest disability in leprosy and occurs in about 10 to 20% of leprosy patients. Various loco-regional flaps have been described for reconstruction of trophic ulcers; however, very large defects are not amenable to local flaps and free flaps form one of the important treatment options. We present a case of a post Hansen's trophic ulcer over the forefoot managed using a radial artery forearm free flap. Debridement of the osteomyelitic bone, removal of the bony prominences, coverage by a well-vascularised tissue, end-to-side arterial anastomosis, use of anterior tibial as the recipient vessel and good postoperative compliance in foot care on the part of the patient gave us good results.

## INTRODUCTION

The term ‘plantar’ ulcer was introduced by Price in 1959.[1] It was defined as a chronic ulceration of the anaesthetic sole of the foot, situated in well-defined areas overlying bony prominences, resistant to local or systemic therapy and characterized by a marked tendency to recurrence.[1] Plantar ulceration is the commonest serious disability in leprosy and the forefoot accounts for 70% of plantar ulcers.[2] Various loco-regional flaps have been described for reconstruction of trophic ulcers; however, very large defects are not amenable to local flaps and free tissue transfer forms one of the important treatment options. Use of free tissue transfer for plantar ulcer in leprosy is sparse in the literature and to the best of our knowledge, this is the first reported case in India. We used a free radial artery forearm flap for coverage of a large post Hansen's trophic ulcer over the distal forefoot and have encountered no recurrence at nine months follow-up.

## CASE REPORT

**Figure 1:**
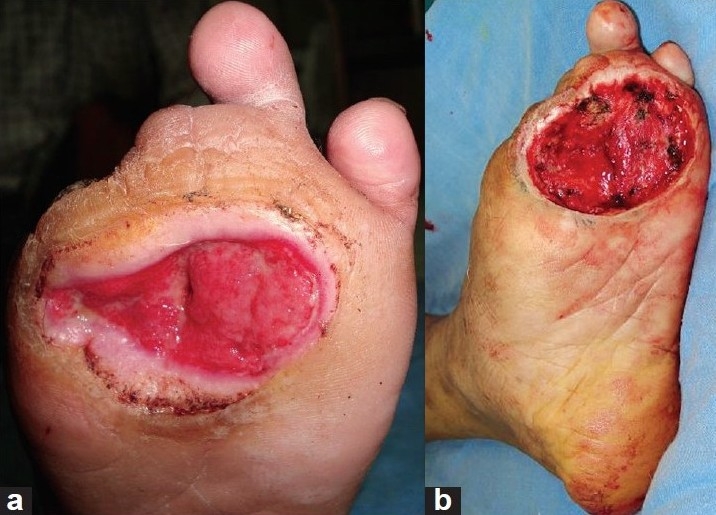
(a) Trophic ulcer before debridement (b) Post-debridement trophic ulcer

**Figure 2:**
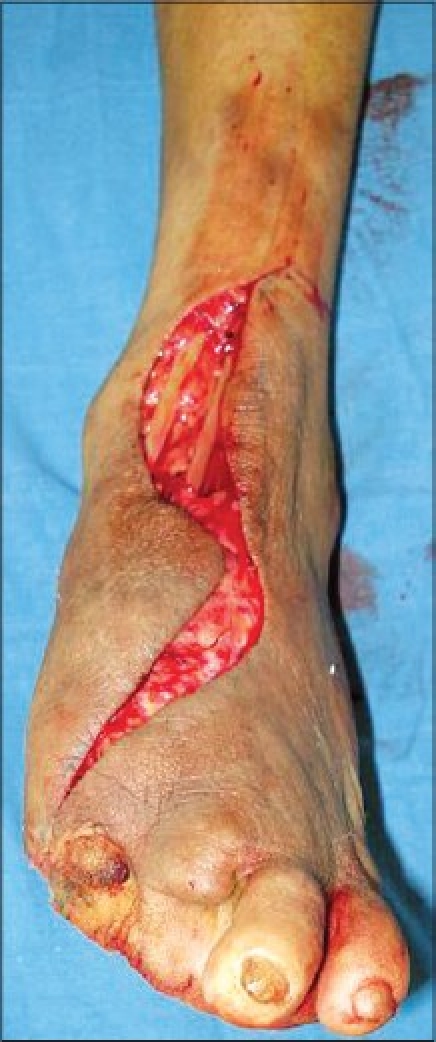
Dissection of the Dorsalis pedis - recipient artery

A middle-aged male, farmer by occupation, presented to us with recurrent trophic ulcer over the forefoot since the past four years. He had completed multi-drug therapy ten years back and was free of active disease. He was managed by various modalities including walking cast, regular dressings, skin grafting and fillet flap from the great toe and second toe at various centres including ours, but had developed a recurrence. He presented to us with a large trophic ulcer over the first, second and third metatarsal head region [Figures 1a and b]. There was a characteristic pencilling and tapering of the distal end of the metacarpal on X-ray. The dorsalis pedis and posterior tibial arteries showed good flow clinically and on handheld Doppler. Sensations were reduced over the entire sole and foot. Debridement of the ulcer was done under tourniquet control; bony prominences and osteomyelitic bone were nibbled off and recipient vessels dissected [Figure 2]. The defect size was 7 cm × 6 cm and local flap was not an option. The pedicle length needed was 13 cm. Considering the defect size and larger pedicle length, the radial artery forearm flap was considered. The flap was raised from the right non-dominant forearm under tourniquet control. End-to-side anastomosis of the radial artery was done to the dorsalis pedis. End-to-end anastomosis of the cephalic vein was done to the venae comitantes. Venous anastomosis was done prior to the arterial anastomosis. The donor area was split skin grafted. The salient intraoperative findings were:

1. The vein was tortuous in nature and was comparatively thin-walled as compared to non-leprosy patients.
2. The intima of the arterial wall was greatly thickened.

**Figure 3:**
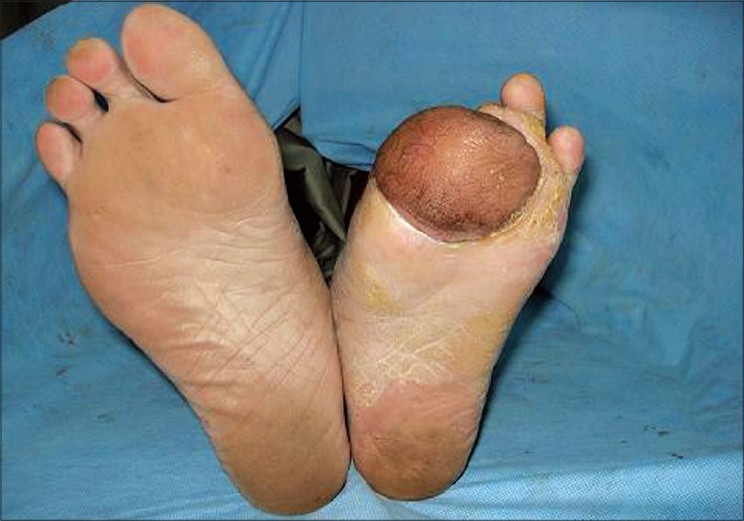
Well-settled flap - plantar view

**Figure 4:**
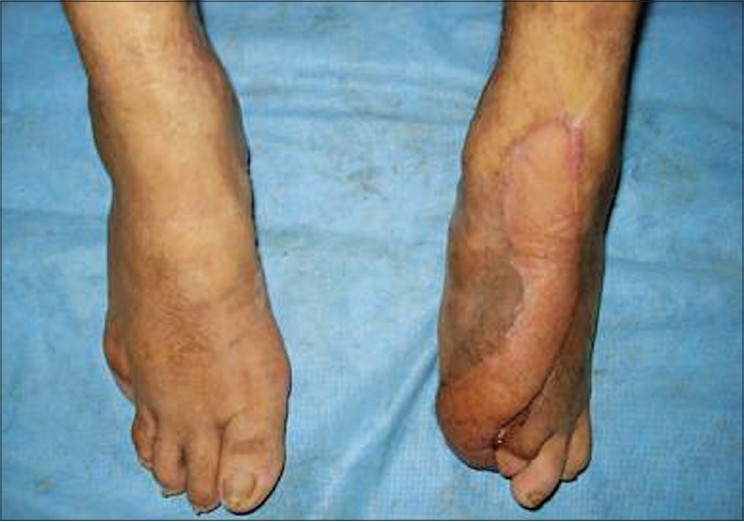
Well-settled flap - dorsal view

After suture removal, the patient was mobilized by gradual weight-bearing from Day 14 after providing protective footwear. The flap was firmly adhered to the undersurface with no difficulty in ambulation. The donor forearm had patchy raw areas, which healed with dressings. The patient was discharged with advice regarding foot care and ambulation, intermittent rest between long stretches of walks and regular use of footwear. After nine months of follow-up there was no recurrence of the ulcer and the flap was well settled [Figures 3 and 4].

## DISCUSSION

Trophic ulcer is the most common complication seen in patients of leprosy, and all the treatment modalities have significant recurrence rates. Plantar ulcers in leprosy are not just defects of tissues like traumatic ulcers. It is a pathological process with loss of tissue, underlying osteomyelitis, loss of or decreased sensation, and decreased blood supply.[3]

Patients managed with rest and dressings have some foci of residual inflammation, and recur soon after the patient is mobilized.[4] For smaller defects, the best method of resurfacing plantar ulcers is use of local tissue. For medium-sized defects plantar artery skin fascia flap by Reiffel,[5] a medial plantar flap with a lateral plantar pedicle by Martin[6] and reverse medial plantar artery flap by Gravem[7] are useful adjuncts. However, the dissection required is meticulous and extensive leaving an equally extensive scarred area and a skin graft over the instep with potential for hyperkeratosis.[5–7]

Larger defects over the forefoot sole region have no availability of local tissue. Cross leg flaps cause increased morbidity in the form of stiffness of joints and potential for formation of bedsore. A Chopart or a Lisfranc amputation[8] can be an option but not many patients are willing for an amputation and cannot be recommended on a routine basis with availability of other options. Free tissue transfer can effectively manage these defects.

Free tissue transfer can provide ample amount of vascularised tissue to the defect. Innervated microvascular flaps are generally not done in leprosy because the recipient nerve is also likely to be involved in the disease process and the flaps have very less chance of even getting protective sensations.[3] Use of end-to-end anastomosis while performing free flaps in leprosy utilizes one of the major limb vessels and has the potential to worsen the already existing condition of ischemia. It is therefore advisable to go for end-to-side anastomosis.

We preferred anterior tibial over the posterior tibial as the recipient vessel because:

1. Posterior tibial is the more dominant vessel of the leg.[9]
2. Anastomosis to the posterior tibial artery would lead to a scar over the weight-bearing region and pressure over the pedicle on early ambulation.

The patient is an integral part of the team, and meticulous, dedicated and lifelong care of the foot on his part is important for good post operative outcome.

Debridement of the osteomyelitic bone, removal of the bony prominences, coverage by a well-vascularised tissue, end-to-side arterial anastomosis, use of anterior tibial vessels as recipient and good postoperative compliance in foot care on the part of the patient gave us good results.
